# Comparative analysis between mandibular positions in centric relation and maximum intercuspation by cone beam computed tomography (CONE-BEAM)

**DOI:** 10.1590/S1678-77572009000700006

**Published:** 2009

**Authors:** Amanda de Freitas FERREIRA, João César Guimarães HENRIQUES, Guilherme Araújo ALMEIDA, Asbel Rodrigues MACHADO, Naila Aparecida de Godoi MACHADO, Alfredo Júlio FERNANDES

**Affiliations:** 1 Undergraduate student, Dental School, Federal University of Uberlândia, Uberlândia, MG, Brazil.; 2 MSc, Dental School, Federal University of Uberlândia, Uberlândia, MG, Brazil.; 3 PhD, Professor, Dental School, Federal University of Uberlândia, Uberlândia, MG, Brazil.; 4 Graduate student, Dental School, Federal University of Alfenas, Alfenas, MG, Brazil.; 5 Graduate student, Dental School, Federal University of Uberlândia, Uberlândia, MG, Brazil.; 6 PhD, Professor, Dental School, Federal University of Uberlândia, Uberlândia, MG, Brazil.

**Keywords:** Computed tomography, Centric relation, Occlusion

## Abstract

This research consisted of a quantitative assessment, and aimed to measure the possible discrepancies between the maxillomandibular positions for centric relation (CR) and maximum intercuspation (MI), using computed tomography volumetric cone beam (cone beam method). The sample of the study consisted of 10 asymptomatic young adult patients divided into two types of standard occlusion: normal occlusion and Angle Class I occlusion. In order to obtain the centric relation, a JIG device and mandible manipulation were used to deprogram the habitual conditions of the jaw. The evaluations were conducted in both frontal and lateral tomographic images, showing the condyle/articular fossa relation. The images were processed in the software included in the NewTom 3G device (QR NNT software version 2.00), and 8 tomographic images were obtained per patient, four laterally and four frontally exhibiting the TMA’s (in CR and MI, on both sides, right and left). By means of tools included in another software, linear and angular measurements were performed and statistically analyzed by student t test. According to the methodology and the analysis performed in asymptomatic patients, it was not possible to detect statistically significant differences between the positions of centric relation and maximum intercuspation. However, the resources of cone beam tomography are of extreme relevance to the completion of further studies that use heterogeneous groups of samples in order to compare the results.

## INTRODUCTION

Centric Relation (CR) is the maxillomandibular relationship in which the condyles articulate with the medial portion of their respective disks, being this complex (disk-condyle) in an antero-superior position against the surface of the articular eminence^1^. The position of maximum intercuspation is defined as the complete intercuspation teeth independent of condylar position.

Numerous studies have been elaborated in order to highlight the possible differences between the position of CR and MI^9^^,^^13^^,^^14^^,^^21^^,^^24^^,^ where different methods have been employed, among these it is possible to highlight studies by imaging. Great emphasis has been given by the scientific community to the Cone Beam Computed Tomography (CBCT)^10^^,^^11^^,^^12^^,^^19^.

Cone Beam Computed Tomography (CBCT) systems have been designed for imaging hard tissues of the maxillofacial region. CBCT is capable of providing sub-millimeter resolution in images of high diagnostic quality, with short scanning times (10–70 seconds) and radiation dosages reportedly up to 15 times lower than those of conventional CT scans. Increasing availability of this technology provides the dental clinician with an imaging modality capable of providing a three-dimensional representation of the maxillofacial skeleton with minimal distortion^12^.

The objective of this study was to assess, quantitatively, in frontal and lateral diagrams, the existence of possible discrepancies in the relation condyle/articulate fossa on the positioning of Centric Relation and on Maximum Intercuspation using the Computed Volumetric Tomography Cone Beam, in asymptomatic young adults presenting normal occlusion and Angle Class I malocclusion.

## SUBJECTS AND METHODS

### Subjects

Ten patients (2 male, 8 female) aged 18 to 25 years were recruited for this study after signing a written informed consent form approved by the Ethics in Research Committee of the Federal University of Uberlândia (n° 479/2008). Within the group of 10 patients, 5 presented normal occlusion (standard) and 5 presented malocclusion, classified as Angle Class I^3^.

The inclusion criteria were: complete natural teething, except for third molars and presence of occlusal contacts with the related antagonist teeth in both arcades. Exclusion criteria were: previous orthodontic handling, presence of non sound teeth, signs of significant periodontal illness, absence of teeth, except for the third molars, symptoms related to prior occlusal adjustment, patients who did not fit the required age pattern for this study (18 to 25 years), patients who wore dental prostheses, patients who presented signs and/or symptoms of temporomandibular dysfunction and facial traumas.

### Procedures

Initially, the clinical survey of patients was carried out, in order to identify the occlusal features of each patient. To register the centic relation, it was performed a manipulation of the mandible and an anterior deprogramming device (JIG) was used. This device was confectioned using acrylic resin chemically activated (Duralay – Reliance Dental Mfg. Co)^16^. In order to maintain a CR position during the tomographic examination, the first contact was identified between superior and inferior arcades in each patient, coinciding with the temporomandibular articulations position, also in CR. Afterwards, a precise wear was performed over the palatal acclivity of the JIG until the obtainment of that first contact which produced a dental stability for the maintenance of both articulations in CR.

#### Computed Tomography

The device utilized in this research was NewTom 3G tomograph (Quantitative Radiology, Summer, Italy) and some parameters were taken into account.

In order to standardize the tomographic image’s inclination orientation on the sagittal view of the primary reconstructions (both in CR and MI), 2 spheres, 5 mm each, were affixed with adhesive tape on each patient. Both spheres were placed following the orientation line of the Frankfurt diagram: the first was placed over the anterior portion of this diagram, that is, on the most inferior point of the left orbital margin (located by means of palpation); the second was placed 3.5 cm posteriorly to the first sphere, bearing the same orientation mentioned on the Frankfurt diagram specifications.

Initially, the first scanning was performed, in which the patient was instructed to keep his/her tooth in maximum habitual intercuspation. This scanning was obtained by means of the device’s specific software (QRNNT, Version 2.00). Afterwards, the second tomographic session was taken when the device of CR maintenance was inserted. At the end of the scannings, data processing was obtained by means of the NewTom 3G’s software in order to achieve the desired images.

On the lateral view of the tomographic images, the inclination of these images was oriented by means of a horizontal line, in tangency with the spheres’ extremity that could be seen, following the Frankfurt diagram specifications. The reconstruction area was standardized superiorly over the fronto-nasal suture, corresponding to the nasion point, and inferiorly, over the most inferior point of the mandible basis. On the frontal view, the inclination was oriented by means of a vertical line that passed internally to the nasal sept until the anterior nasal spine. There were used 0.2 mm tomographic slices, which made possible the secondary reconstructions of the images.

The measurements on the sagittal and frontal norms were carried out bilaterally on both mandibular positions in Centric Relation and Maximum Intercuspation, according to the following images:

1– Greater medium-lateral dimension of the condyles: axial image in which the condyle has shown the greatest medium-lateral linear dimension, measured until the tangency of its external cortical walls.
2– TMA lateral image selection: image obtained by means of an angle tool to orientate perpendicular cuttings to the greatest mediumlateral dimension of the condyle head.
1– TMA frontal image selection: image obtained by means of an application of a cut coincident to the condylar dimension on an axial view.

**Figure 1:**
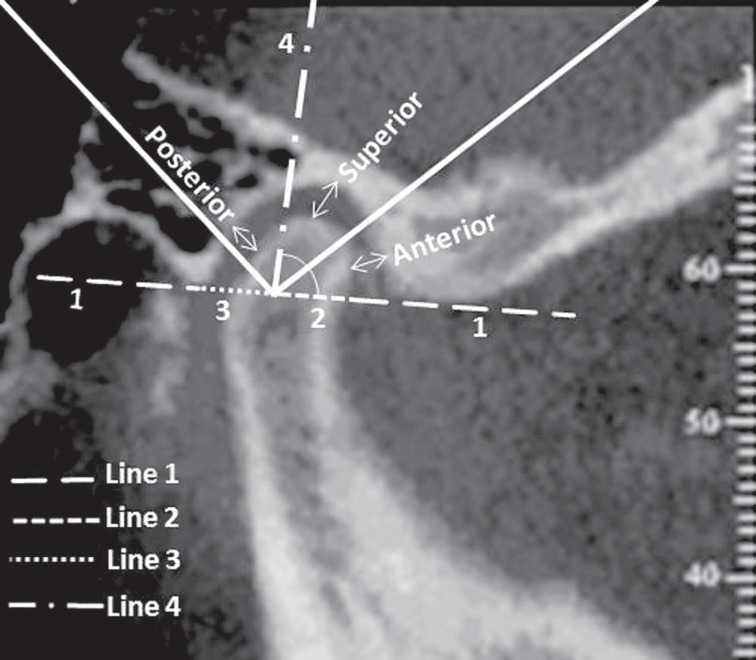
Lateral view of the distances of the condyle/articular fossa, over the posterior, superior and anterior senses

On the lateral cuttings, there were assessed the distances of the relations condyles/articular fossa, over the posterior, superior and anterior senses, according to the following mensuration standards (Figure 1):

- Line 1: Reference line, joining the most inferior points of the posterior region of the fossa and of the articular eminence, in order to measure the lateral cuttings.
- Line 2: Extension on which the Line 1 overlapped the condylar process, on the antero-posterior sense.
- Line 3: Line regarding the demarcation of the medium point on Line 2.
- Line 4: From the adaptation of the 90º tool, positioned exactly on the medium point of the reference, an extension of the vertical rod of this tool was performed in order to obtain a linear measurement, that means the distance between the medium point of the Line 2 and the most superior point of the cortical wall of the condyle head bone.

By means of the vertical rod of the angle tool included into the device's software, there were established the desired posterior, superior and anterior measurements on the lateral images.

**Figure 2:**
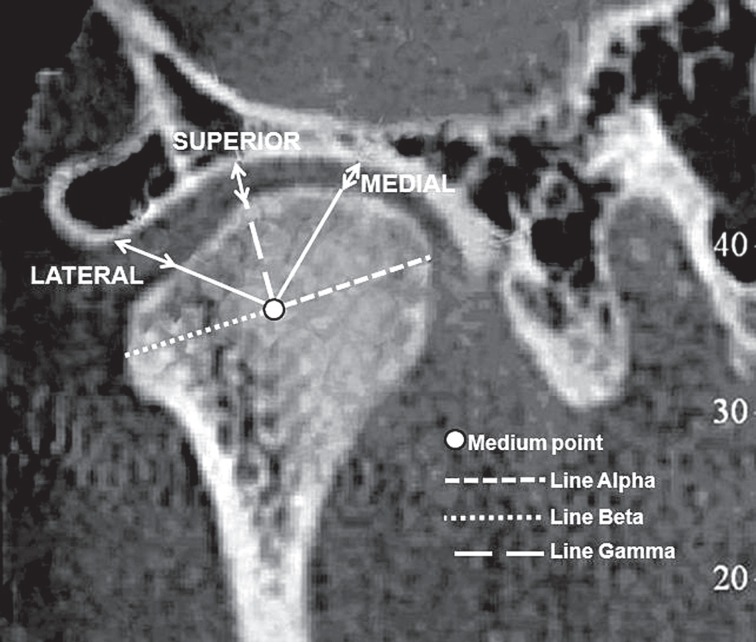
Frontal view of the distances of the condyle/articular fossa, over the posterior, superior and anterior senses

On the frontal cuttings, the measurements comprised the distances between the condyles and the external surface of the articular fossa, on the medial, superior and lateral senses. On the methodology of the frontal cuttings, the initial references were found directly over the condyles head, where the most lateral and medial points were identified, by means of the angle tool. Since then, there were standardized the following reference lines (Figure 2):

- Line Alpha: identifying the most medial and lateral point over the condyle head
- Line Beta: drawn overlapping the alpha line, until reaching its middle length, determining the medium point of reference
- Line Gamma: extending from the medium point of reference until the most superior point of the external cortical wall of the condyle head, aiming to certificate the encounter of this same medium point of reference on the frontal right cuttings in RC and MI.

By means of the movement of the vertical rod of the angle tool, there were established the superior, medial and lateral measurements. On the same manner, the identification of the lines alpha, beta, gamma and the medium point of reference was performed.

The non-coincidence of the values of the standardized lines at this present study was interpreted as two different medium points of reference on 2 positions. If any coincidence was noticed, all the methodology had to be restarted.

### Statistical Analysis

After the obtainment of all the measurements, the average and standard deviation were established for each one of them, following their respective positioning and corresponding cuttings, for the application of the statistical tests: the method’s Error Test, student t test and Tukey tests at a 5% level of significance.

### Intra-examining error test:

A unique user, previously calibrated, carried out the trial selection and measurement of the images of interest for this work. This user applied the Test of Intra-Examining Error after 20 past days of all the measurement carried out by him. 5 patients and 3 measures of the research were randomly chosen for the statistical accomplishment of the Error Test. The new measurements were remade using the software Basic 3G, without the knowledge of the values of the measures initially found (annex 5). Of possession of the values of the measures that were remade, comparisons between the average of the initial measurements with those performed 20 days after the first ones were carried out. Therefore, the T of Student test shows the results on Table 1.

**Table 1:** Comparisons among the means obtained by the student t test, for evaluation of eventual differences between maximal intercuspation (MI) and centric relation (CR)

Variable		Mean	Standard deviation	t-Student	p-value
Lat right POST	MI	1.88	0.529	1.303	0.209
	CR	1.61	0.387		
lat right ANT	MI	1.76	0.744	-0.892	0.384
	CR	2.07	0.808		
lat right UPPER	MI	2.41	0.810	0.093	0.927
	CR	2.38	0.625		
fron right LAT	MI	2.32	0.789	-0.307	0.763
	CR	2.43	0.815		
fron right UPPER	MI	2.81	0.736	0.244	0.810
	CR	2.73	0.729		
fron right MED	MI	2.75	0.940	0.189	0.852
	CR	2.67	0.955		
lat left POST	MI	1.89	0.536	0.940	0.360
	CR	1.67	0.510		
lat left ANT	MI	1.88	0.721	0.034	0.973
	CR	1.87	0.581		
lat left UPPER	MI	2.69	0.828	0.749	0.464
	CR	2.43	0.721		
fron left LAT	MI	2.47	0.648	0.247	0.808
	CR	2.40	0.620		
fron left UPPER	MI	3.01	0.722	0.318	0.754
	CR	2.90	0.821		
fron left MED	MI	2.85	0.905	0.142	0.889
	CR	2.79	0.987		

P-value > 0.05: not statistically significant differences between the mean of MI and CR, accomplished by the student t test.

## RESULTS

Tables 1, 2 and 3 show the results of comparison between two positions: RC and MI. The standard deviation of each position for every patient was calculated considering, independently, the left and right sides. The averages between the standard deviation for CR and MI standard deviation for both left and right sides were calculated. The results of the test error of intra-examiner has not shown significant differences.

**Table 2:** Comparisons between the means obtained by the student t test, for evaluation of the eventual differences within Group I, regarding maximal intercuspation (MI) and centric relation (CR)

Variable		Mean	Standard deviation	t-Student	p-values
Lat right POST	MI	1.820	0.5718	0.524	0.614
	CR	1.640	0.5128		
lat right ANT	MI	1.680	0.6458	-0.762	0.468
	CR	2.000	0.6819		
lat right UPPER	MI	2.360	0.7403	-0.269	0.795
	CR	2.480	0.6686		
fron right LAT	MI	2.080	0.7981	-0.121	0.907
	CR	2.140	0.7701		
fron right UPPER	MI	2.660	0.5505	0.367	0.723
	CR	2.540	0.4827		
fron right MED	MI	2.860	0.9397	0.155	0.881
	CR	2.760	1.0922		
lat left POST	MI	1.960	0.6269	0.202	0.845
	CR	1.880	0.6261		
lat left ANT	MI	1.540	0.8473	-0.128	0.902
	CR	1.600	0.6205		
lat left UPPER	MI	2.84	0.727	0.581	0.577
	CR	2.60	0.570		
fron left LAT	MI	2.56	0.456	0.553	0.595
	CR	2.42	0.335		
fron left UPPER	MI	3.060	0.6427	0.414	0.690
	CR	2.880	0.7294		
fron left MED	MI	2.880	1.2215	0.026	0.980
	CR	2.860	1.1929		

P-value > 0.05: not statistically significant differences between the mean of MI and CR obtained by the student t test.

**Table 3:** Comparisons among the means obtained by the student t test, for evaluation of the eventual differences within the Normal Group regarding maximal intercuspation (MI) and centric relation (CR)

Variable		Mean	Standard deviation	t-Student	p-value
Lat right POST	MI	1.940	0.5413	1.332	0.219
	CR	1.580	0.2683		
lat right ANT	MI	1.840	0.9017	-0.499	0.631
	CR	2.140	0.9965		
lat right UPPER	MI	2.460	0.9607	0.349	0.736
	CR	2.280	0.6380		
fron right LAT	MI	2.560	0.7861	-0.313	0.763
	CR	2.720	0.8319		
fron right UPPER	MI	2.960	0.9263	0.068	0.947
	CR	2.920	0.9338		
fron right MED	MI	2.64	1.036	0.097	0.925
	CR	2.58	0.915		
lat left POST	MI	1.82	0.492	1.412	0.196
	CR	1.46	0.288		
lat left ANT	MI	2.220	0.4025	0.300	0.772
	CR	2.140	0.4393		
lat left UPPER	MI	2.54	0.979	0.476	0.647
	CR	2.26	0.879		
fron left LAT	MI	2.380	0.8468	0.000	1.000
	CR	2.380	0.8672		
fron left UPPER	MI	2.960	0.8678	0.068	0.948
	CR	2.920	0.9910		
fron left MED	MI	2.820	0.5891	0.213	0.837
	CR	2.720	0.8701		

P-value> 0.05: not statistically significant differences between the means of MI and CR by the student t test.

**Table 4:** Estimates of means of variables lateral right POST, lateral right ANT, lateral right SUP, frontal right LAT, frontal right SUP, frontal right MED, lateral left POST, lateral left ANT, lateral left SUP, frontal left LAT, frontal left SUP, frontal left MED, cutting in the maximal intercuspation and centric relation

Variable	Grupos	
	Class I Angle	Normal
Lat right POST	1.73 a	1.76 a
	1.84 a	1.99 a
lat right ANT	2.42 a	2.37 a
	2.11 a	2.64 a
lat right UPPER	2.60 a	2.94 a
	2.81 a	2.61 a
fron right LAT	1.92 a	1.64 a
	1.57 a	2.18 b
fron right UPPER	2.72 a	2.40 a
	2.49 a	2.38 a
fron right MED	2.97 a	2.94 a
	2.87 a	2.77 a

Proportions followed by the same letter are not statistically different by the test Tukey, considering a level of significance of 0.05.

## DISCUSSION

A mandibular position that determines occlusal, muscular and articular balance is indispensable to plan and execute oral rehabilitation, in concordance to the stomatognathic system. There is little consensus on the contours of concept of Centric Relation as a reference position for oral rehabilitation^5^^,^^7^^,^^16^^,^^17^^,^^23^^,^^25^^,^^26^. A fundamental question in dentistry is what is the optimal position of the condyle in the articular fossa when teeth are in maximum intercuspation. Despite the way the teeth come together in occlusion can be observed directly in the patient’s mouth, the condylar position into the articular fossa is impossible to be seen by the clinician’s naked eye.

In order to estimate the condylar positions, several methodologies have been proposed at the current literature. However, all those methodologies have shown many controversies. Within the most used methods to measure different mandibular positions, it can be taken into account the use of semi-adjustable articulators and imaging examinations^2^^,^^5^^,^^7^^,^^8^^,^^9^^,^^13^^,^^14^^,^^21^^,^^24^. According to the semi-adjustable articulators use, some drawbacks can be highlighted when treating and/ or studying the condylar positions, i.e.: the fact of the articulators not considerate the presence and anatomical variability of the present tissues at the temporomandibular articulation and possible distortions during the assemblage on these apparatuses.

Various radiographic modalities have been used to visualize the condylar positions. In spite of that, the radiographies obtainment represents a non-precise method for this analysis, for the various magnificence radiographic degrees and, also, because there is a restriction related to the two-dimensional diagram. Another important imaging resource is the magnetic resonance, a very applied method along the clinical studies, which refers to the articular disc positioning. Despite this study has not employed the magnetic resonance method, attention can be called to the association of magnetic resonance technology and computerized tomography - techniques of great contribution to a better comprehension of the temporomandibular area.

Within the imaging resources, this study employed the cone beam computed tomography technology, considering that the spatial variations of the condyles related to the fossa on RC and MI positions are predominantly very small. This method allows a three-dimensional assessment of the temporomandibular articulation, presenting higher precision over images delineation. Within the imaging exams, TCBT presents more substantial details when compared to other exams, allowing greater trustworthiness from the acquired data. CTCB presents lower radiation levels and lower costs when compared to the conventional computed tomographs of the medical area^11^^,^^20^^,^^21^^,^^22^.

Several researchers have related that the majority of toothed patients exhibit a discrepancy between the CO and MI positions^9^^,^^13^^,^^14^^,^^21^^,^^24^. According to the results of this study, the analyzed patients presented differences between these two positions, however, when submitted to a statistic analysis, those differences were not significant. Within the possible explanations for this result, the features of the selected sample can be highlighted, constituted by young and asymptomatic adult individuals. The use of a reduced sample of 10 individuals could be explained for the pilot character of this type of study, employing the cone beam method, and because it is a research which deals with x-radiation directly over human beings.

Most of the studies that demonstrated a significant discrepancy between RC and MI used heterogeneous samples, in which were included patients of different ages, with symptoms of TMD and absence of occlusal stability^9^^,^^13^^,^^14^^,^^21^^,^^24^. Because of these differences, it could be presumed that, in spite of the samples presented various occlusal arrangements, these were in relative balance or were not yet capable of generating alterations that could create significant changes over the condyle/fossa relation.

The results obtained in this present study are of clinical relevance. It is indispensable to carefully examine the status of the condyles and discs while performing three-dimensional occlusal reconstruction by orthodontic, prosthodontic or other modalities in dentistry. In spite of the great importance of differentiation of these two condylar positions in any dentistry modality and of the results achieved in this study, attention must be given to the fact that each patient has unique features that should be carefully examined in order to obtain a suitable result.

Further studies are needed in this research area. Studies which involve a greater amount of patients (samples) with normal patterns of occlusion, without parafunctional habits, asymptomatic vs. symptomatic, young patients vs. old patients, dentulous vs. edentulous and many more variables that could reveal information for the establishment of some parameters, yet obscure, taking into account the worthiness of the cone beam method.

## CONCLUSION

Based on the limitations of the present study, it was concluded that there are not statistically significant differences between centric relation and maximum intercuspation in the group of individuals without any symptom or sign of temporomandibular disorder.
